# Activity and connectivity changes of central projection areas revealed by functional magnetic resonance imaging in Na_V_1.8-deficient mice upon cold signaling

**DOI:** 10.1038/s41598-017-00524-x

**Published:** 2017-04-03

**Authors:** C. Heindl-Erdmann, K. Zimmermann, P. Reeh, K. Brune, A. Hess

**Affiliations:** 1 Friedrich-Alexander University Erlangen-Nürnberg (FAU), Institute of Experimental and Clinical Pharmacology and Toxicology, Fahrstraße 17, 91054 Erlangen, Germany; 2 Friedrich-Alexander University Erlangen-Nürnberg (FAU), Department of Anaesthesia, University Hospital Erlangen, Krankenhausstraße 12, 91054 Erlangen, Germany; 3 Friedrich-Alexander University Erlangen-Nürnberg (FAU), Institute of Physiology und Pathophysiology, Universitätsstraße 17, 91054 Erlangen, Germany

## Abstract

The voltage-gated sodium channel subtype Na_V_1.8 is expressed in the peripheral nervous system in primary afferent nociceptive C-fibers and is essential for noxious cold signaling. We utilized functional magnetic resonance imaging on Na_V_1.8-deficient (Na_V_1.8^−/−^) compared with wildtype (WT) mice to identify brain structures decoding noxious cold and/or heat signals. In Na_V_1.8^−/−^ mice functional activity patterns, activated volumes and BOLD signal amplitudes are significantly reduced upon noxious cold stimulation whereas differences of noxious heat processing are less pronounced. Graph-theoretical analysis of the functional connectivity also shows dramatic alterations in noxious cold sensation in Na_V_1.8^−/−^ mice and clearly reduced interactions between certain brain structures. In contrast, upon heat stimulation qualitatively quite the same functional connectivity pattern and consequently less prominent connectivity differences were observed between Na_V_1.8^−/−^ and WT mice. Thus, the fact that Na_V_1.8^−/−^ mice do not perceive nociceptive aspects of strong cooling in contrast to their WT littermates seems not only to be a pure peripheral phenomenon with diminished peripheral transmission, but also consists of upstream effects leading to altered subsequent nociceptive processing in the central nervous system and consequently altered connectivity between pain-relevant brain structures.

## Introduction

Evolutionary pressure requires nociceptive processing functions through the entire range of noxious temperatures from hot to cold to enable protection of the organism from hazardous tissue damage. Previously the sodium channel Na_V_1.8 was shown to be responsible for the continued excitability of nociceptors in noxious cold conditions because of its specialized inactivation properties. Its ablation results in a cold resistant phenotype in mice reported and validated from different laboratories^1, 2^. Na_V_1.8 is expressed especially in peripheral sensory neurons as well as in small and medium-sized DRG neurons and their axons^3–5^ and in at least 75% of slowly conducting C-fibers in the peripheral nervous system^1, 6^. Thus, high levels of the tetrodotoxin-resistant channel (equivalent to Na_V_1.8) were detected in sensory but not in central neurons^7^.

In the past, several functional imaging studies addressed the question of which brain regions are uniquely required for and presumably activated during the perception of pain in humans^8, 9^. Recent research, however, uncovered that the identified areas were not only specifically related to nociceptive processing but process salient signals originating from multisensory input rather than to generate the feeling of pain alone^10, 11^. A functional magnetic resonance imaging (fMRI) study on human subjects dealing with noxious heat (46 °C) and cold (5 °C) stimulation ruled out that the patterns of brain activation upon noxious heat and cold stimulation were quite common^12^. Significant differences of activation properties between hot and cold conditions were detected in prefrontal areas^12^. The impact of the sodium channel Na_V_1.8 on cold and heat nociception, however, was not addressed in this study. More recently, imaging studies were also performed in rodents, which showed surprisingly consistent results with human brain imaging studies, in regard to quite similar activation patterns^13, 14^. In particular, a pattern of activated areas in the medial and lateral pain system was detected upon nociceptive processing^13, 14^. Therefore, these findings demonstrate the potential of functional imaging for translation of findings from mice to humans.

In this context we sought to utilize functional magnet resonance imaging (fMRI) in combination with genetically modified mice as a versatile combination to study functions of specific genes/proteins within central processing of noxious input information^15^. Specifically, we focused on the impact of a lack of the voltage gated sodium channel Na_V_1.8 on the cerebral manifestation of noxious cold and heat temperatures to identify the related brain structures and their interactions contributing to the perception of cold and heat noxious input.

Previous behavioural thermal pain tests on Na_V_1.8-deficient (Na_V_1.8^−/−^) mice had revealed a strongly attenuated sensitivity to cold in the cold plate test compared to the wildtype (WT)^2^ and a mildly reduced sensitivity to heating in the Hargreaves but slightly increased sensitivity in the hot plate test^1, 7^. Along this we applied noxious cold (0–20 °C) and heat (40–55 °C) stimulation to the right dorsal hind paws of Na_V_1.8^−/−^ mice and their WT littermates and simultaneously measured blood oxygenation level dependent (BOLD) fMRI.

## Methods

### Experimental Animals

The care and use of animals was conformed to the national guidelines. All experimental protocols were carried out in strict accordance with the recommendations of the Guide for the Care and Use of Laboratory Animals of the National Institutes of Health and the relevant guidelines and regulations concerning animal experiments, i.e. the protocol for *in-vivo* experiments in animals was reviewed by the local animal ethics committee (Committee on the Ethics of Animal Experiments of the Friedrich-Alexander Universität Erlangen-Nürnberg) and approved by the local district government (Regierung Mittelfranken, Permit Number: 54-2532.1-29/10).

Two experimental series were performed on 10–14 weeks old male healthy Na_V_1.8^−/−^ and WT-mice of the C57/BL6 strain weighting between 20 and 25 g and bred at the Department of Physiology and Pathophysiology, Erlangen as for the previous paper^2^, where all details of the mouse strain and details on their behavioural assessments were already published. Therefore, behavioural experiments were not duplicated for the recent approach, particularly in support of the 3R concept of refinement and reduction of animal experiments^16^. From the initially 13 measured animals per group 10 animal data sets per group were selected for further analysis, 3 animals per group were excluded due to strong motion artifacts caused by irregular breathing. This resulted in n = 10 animals per group. Each mouse was used for both, cold and heat stimulation, at the laboratory of the Institute of Experimental and Clinical Pharmacology and Toxicology, Friedrich-Alexander University Erlangen-Nürnberg (FAU). Delay between experiments was minimum 3 days, 7.4 ± 2,3 days for the Na_V_1.8^−/−^ group and 5,2 ± 2,8 days for the WT group. A light/dark cycle was pretended simulating natural conditions. Food and water were given ad libitum. The fMRI preparation (mounting of the animal, basic adjustments and MRI reference measurements) took 30–40 min, a time in which also a constant physiological pCO_2_ level (around 38 mmHg ± 10%) was reached^17^. The anaesthesia of the mice was induced with medical air plus 5% isoflurane in a vaporisator for 7 min. The animals were placed on a cradle and fixed by a bite bar. A dedicated surface coil (Rapid mouse surface coil, Rapid Biomedical GmbH, Rimpar, Germany) was mounted and the animals were then moved inside the MR scanner (BRUKER, Ettlingen, Germany). During the whole mounting and the following MRI session a respiration rate around 100 ± 20 BPM and therefore a constant pCO_2_ level was achieved by slight adjustments of the isoflurane supply (around 1.2%). Respiration rate was measured constantly by a custom made monitoring system using a pressure sensitive breath sensor. Moreover, the body temperature of each animal was maintained at a constant level of 37 °C using a warm water circuit inside the cradle. After the end of the last experiment the animals were killed according to the animal license by isoflurane overdoses and CO_2_.

### Imaging data acquisition

The MRI experiments were performed on a 4.7 T animal MR BRUKER BioSpec scanner 47/40 (BRUKER, Ettlingen, Germany) with a 40 cm horizontal free bore. A whole-body birdcage resonator operating with an actively shielded high-power gradient system (200 mT/m) enabled homogenous excitation. The low-noise, actively RF-decoupled and anatomically shaped 2 × 2 phased array head coil was used to receive the signals. No ear bars were used during the experiment, because ear bars are a confounding painful stimulus, even under light anaesthesia.

Firstly, a 1 min single slice echo planar imaging (EPI) MR measurement (64 × 64 matrix, TR = 200 ms, TEef = 24.4 ms, 300 repetitions) was acquired and run as a movie to control the stability of the head fixation. In case of visible movements above 1 pixel the animal was remounted.

Functional BOLD MRI scans were performed using a T2*-weighted single-shot gradient echo based EPI. A functional scan consisted of 750 acquisitions of 22 axial slices (EPI, 64 × 64 matrix, TR = 2000 ms, k-space averaging of 2, resulting in total TR of 4000 ms (needed for covering 22 slices), TEef = 24.4 ms, FOV 15 × 15 mm, in-plane spatial resolution 234 × 234 μm, slice thickness 500 μm). Using this sequence and our optimized phased array coil we were able to achieve a temporal signal-to-noise ratio^15, 18, 19^ of 55.5 ± 10 for cortex and 53.66 ± 11.84 for thalamus. Thereafter an anatomical reference scan was performed at slice positions identical to the functional ones by a fast spin echo sequence (RARE, 256 × 256 matrix, TR = 3000 ms, RARE factor = 8, TEef = 47.1 ms, FOV 15 × 15 mm, NEX = 10, slice thickness 500 μm)^20^.

### Thermal stimulation

During each of the two 50 min fMRI experiments either a set of only contact cold stimuli of increasing temperature (0 °C, 5 °C, 10 °C, 20 °C ± 1 °C, 15 s of ramp-shaped cooling followed by a 5 s plateau) or only warm/heat stimuli (40 °C, 45 °C, 50 °C, and 55 °C, ± 1 °C, plateau for 5 s after 15 s of heat increase), triggered by the MR, were applied using a customized feed-back controlled Peltier element (Kryotherm, Saint-Petersburg, Russia) attached to the dorsal surface of the right hindpaw (allowing more homogenous temperature exposure to the skin compared with the horny plantar side). Mixed experiments providing alternating cold and heat stimuli in one experiment were not investigated. The individual stimuli were presented at intervals of 3 min 25 s and the whole set of stimuli was repeated three times. This interval ensured that the baseline was reached between stimuli as validated by the time courses inspected. The stimulation device did not interfere with the magnetic field of the MR scanner. We chose a cross over design, meaning that the chronological order of the complete either heat or cold stimulation experiment was varied through the animals in order to avoid systematic bias due to the stimulation order.

### Data processing

The functional data were analyzed using Brain Voyager QX (V 2.6, Brain Innovation B.V. Maastricht, Netherlands) and MagnAn 2.3, (BioCom GbR, Uttenreuth) an IDL6.4 application (Exelis Visual Information Solutions, Boulder, CO, USA)^13, 21^ (for overview of the analysis workflow also see Supplementary Figure S1). In brief, pre-processing of the data was performed in Brain Voyager QX using the following steps: Motion correction using rigid registration and resampling with sinc interpolation, slice time correction performed with a Cubic Spline, linear detrending, high pass filtering (9 cycles) and temporal smoothing (12 seconds), spatial 2D Gaussian smoothing of the data (FWHM kernel: 2 pixel, in-plane direction). The motion detected for the included 10 animals of each group, was always below 1 pixel. Next a general linear model analysis (GLM) of the time series data with each temperature as separate predictor was calculated. Subsequently for the following combinations of the predictors (i.e. contrasts) statistical values (t-values) per voxel were calculated: each temperature separately, all combined cold and all combined heat temperatures. These contrasts can be visualized as activation maps, called statistical parametric maps (SPMs). To identify stimulus dependent significantly activated voxels SPMs of all contrasts were generated for all individual animals. All SPMs were corrected for multiple comparison using false discovery rate (FDR, q = 0.05, dependent, two-sided) and thresholded to detect significantly activated voxels.

The first volume of the pre-processed time series data was used for segmentation. Segmentation was performed manually in AMIRA (FEI, Hillsboro, USA) and the resulting binary brain mask was used to cut out the brain. In order to perform a group-wise analysis of significantly activated voxels all animals had to be registered to a reference subject. Registration was performed by an affine transformation scheme (six degrees of freedom) using the grey value information of each individual segmented brain. As reference the subject was chosen which led to the overall lowest variance registering all datasets to that reference. The registered datasets were averaged to create an anatomical template for subsequent labeling of certain brain structures (in total 196). For that purpose we used a digital 3-dimensional brain atlas which was generated in our lab based on digital outlines of the brain regions from the commercially available mouse brain atlas from Paxinos (third edition)^22^. This 3D atlas was manually registered to the average anatomical template. Via inverting the subject specific registration matrix this atlas was transformed back to the individual first volumes and consequently also to the individual SPMs and the whole time-series data. Next, for each brain structure per animal the significantly activated voxels of the thresholded contrast specific SPMs were labelled using the individually registered digital atlases (see above). A minimum cluster size of 4 voxels was set for defining activated brain volumes. Thus, these significantly activated voxels can be counted as the activated volume per given brain structure.

**Figure 1:**
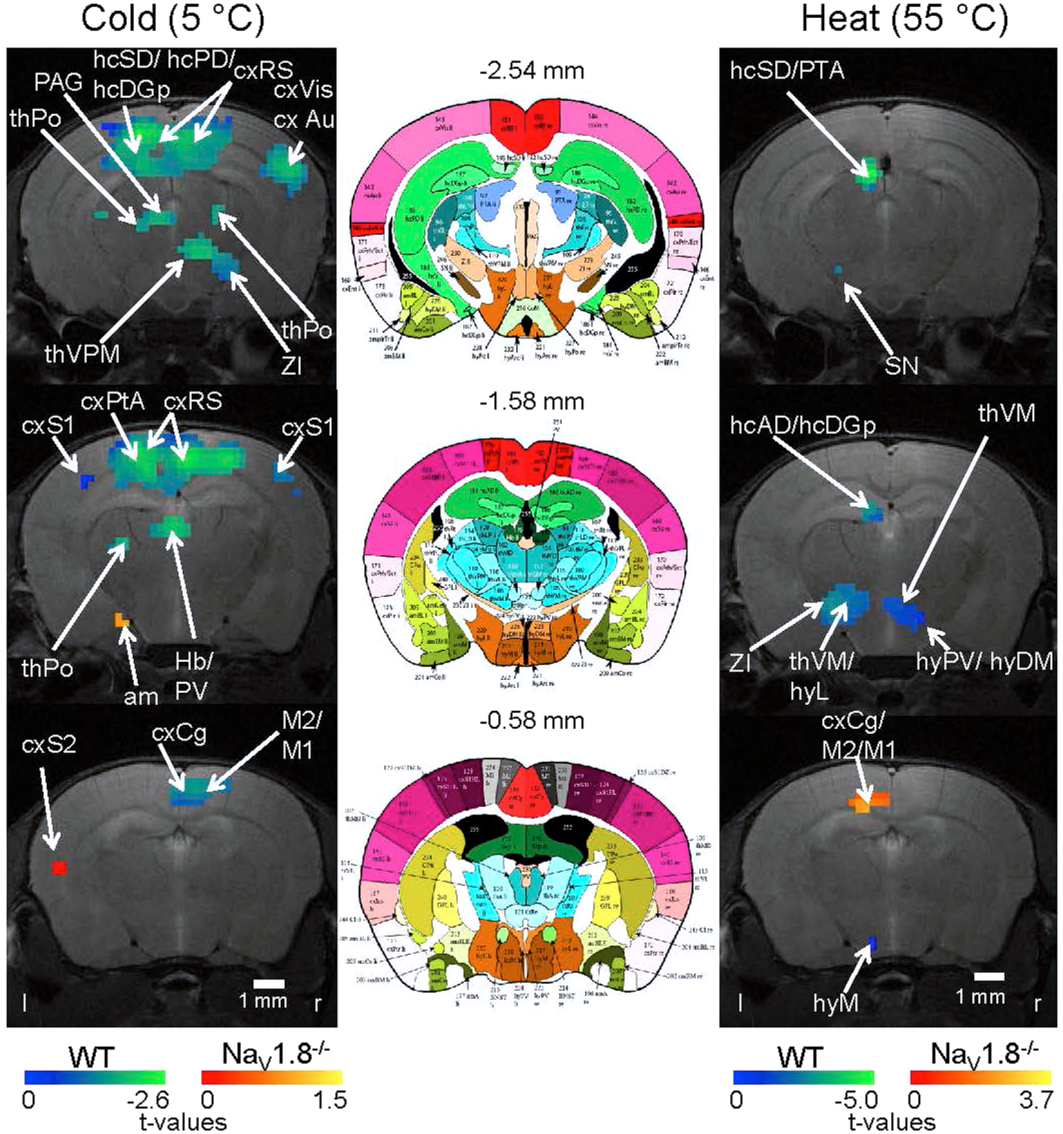
Second order statistics of activation maps of Na_V_1.8^−/−^ and WT mice following cold and heat stimulation. Activation maps show differences in t-values (Na_V_1.8^−/−^ and WT, see Materials and Methods) at voxels which are significantly different between WT and Na_V_1.8^−/−^ mice upon cold (5 °C, left column) and heat (55 °C, right column) stimulation. The negative values (blue-to-green scale) indicate stronger activation in WT, the positive values (red-to-yellow scale) indicate stronger activation in Na_V_1.8^−/−^. Arrows point to brain structures which were identified by matching with the digital atlas (middle column). The numbers above the atlas slides indicate the respective bregma coordinate. For abbreviations see Supplementary information.

In order to visualize the spatial distribution of significant differential brain activity between Na_V_1.8^−/−^ and WT mice upon either heat (55 °C) or cold (5 °C) stimulation conditions a voxel-wise second order statistic (homoscedastic Student’s t-test; two-sample) based on the average SPMs per group was calculated (Fig. 1). The resulting average p-value map was thresholded at p < 0.15 to obtain a binary mask encoding a wide range of (significant) differences. Next, the average WT-SPM per stimulus was subtracted from the corresponding SPM of the Na_V_1.8^−/−^ group and the resulting differences (t-values larger or lower than 0) were multiplied with the binary mask. The resulting data were visualized in AMIRA on top of the corresponding anatomical MRI scans. The negative values, blue-to-green scale, indicate stronger activation in WT and the positive values, red-to-yellow scale, indicate stronger activation in Na_V_1.8^−/−^ (Fig. 1).

**Figure 2:**
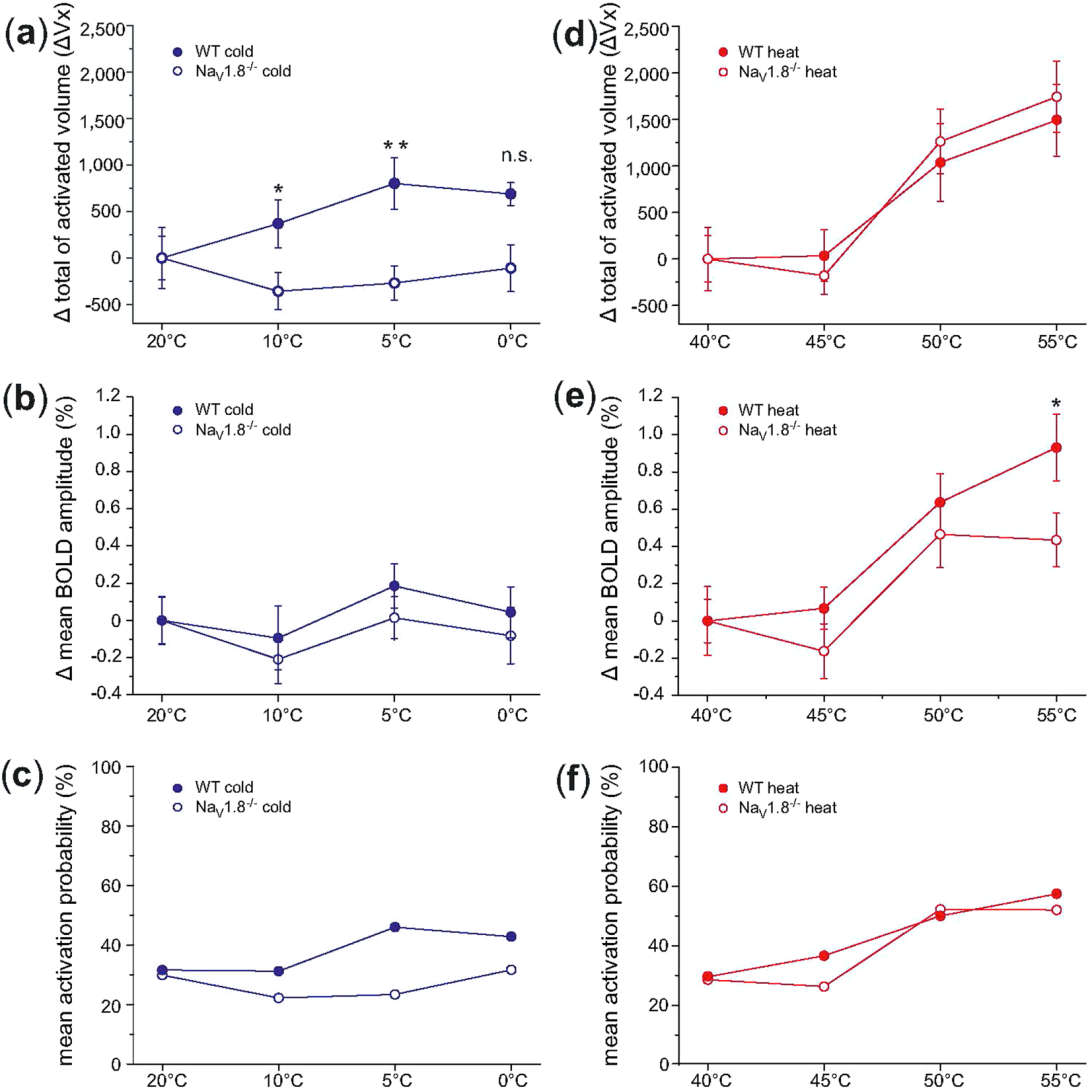
Brain-wide quantification of activated volume, mean BOLD amplitude changes and mean activation probability upon cold and heat stimulation conditions for Na_V_1.8^−/−^ and WT mice. Stimulus-response curves were calculated from the functional data set of both genotypes (WT: •, Na_V_1.8^−/−^: ⚬) and both stimulation conditions (cold: blue, 20 °C to 0 °C and heat: red, 40 °C to 55 °C) for average activated volumes (Vx stands for Voxel) (cold: (**a**), heat: (**d**)), mean BOLD amplitudes (cold: (**b**), heat: (**e**)) and mean activation probabilities (cold: (**c**) and heat:(**f**)). Data are presented as mean values across animals and all brain structures for each stimulation temperature referenced to 20 °C as baseline in case of cold stimulation and non-noxious 40 °C in case of heat stimulation, respectively. Brain structures, which were not significantly activated, were included with 0 in the averaging process, which leads to the low mean activation probabilities. Data are presented as mean values ± SEM, n = 10 for WT and Na_V_1.8^−/−^, respectively. Significant differences between Na_V_1.8^−/−^ and WT mice for each stimulation condition are marked with asterisks: **p ≤ 0.01; *p ≤ 0.05; n.s. not significant; Student’s t-test, two-sample; multiple comparison correction with α ≤ 0.05.

Furthermore, for each brain structure, animal and contrast, the time series of all activated voxels were averaged and an average time profile (atp) of each stimulus temperature was created. These atp’s were used to determine the stimulus-induced change of the mean BOLD amplitude. Additionally, the mean activation probability (probability of brain structure activation across subjects) was calculated. To provide a whole brain group comparison between WT and Na_V_1.8^−/−^ mice for the different temperatures, the parameter values were averaged across all animals and activated brain structures. In order to provide a better comparability, the activation values of the non-noxious stimulation (cold: 20 °C and heat: 40 °C) served as reference values and were subtracted from the values of all the other stimulation temperatures (Fig. 2). A Student’s t-test (two-sample; p ≤ 0.05; n = 10 for WT and Na_V_1.8^−/−^, respectively) was performed to show significant differences between WT and Na_V_1.8-deficient mice for the different parameters.

For connectivity analysis the average time courses of the significantly activated voxels of each brain structure were corrected for global signal fluctuations by linear regression. The residual time courses of all brain structures were cross-correlated separately for each stimulus predictor and animal. Next, the individual cross-correlation matrices for the cold and heat predictors were averaged over all animals resulting in averaged cross-correlation matrices. In order to compare the number of connections across stimulation condition and genotype the average matrices where binarized using FDR threshold (q = 0.05, dependent). Significant differences between the matrices of the two genotypes (Na_V_1.8^−/−^ and WT) and the two stimulation conditions (cold and heat, all temperatures) were calculated using a homoscedastic Student’s t-test (two-sample). Significant different connections per stimulation condition were determined by thresholding the difference matrices at p < 0.05. The resulting binary adjacency matrices were transformed into graphs with brain structures as nodes and significant differences as edges. The weight of the edges was −1 for negative differences and +1 for positive ones. These networks were visualized in a custom made module in AMIRA. The 3D coordinates of each brain structure – node – were taken from our 3D mouse brain atlas derived from Paxinos and Watson^22^ and nodes without edges (orphan nodes) were omitted. The node-size represents its number of edges, i.e. significantly different connections with other brain structures across the whole brain. For a better appreciation of the 3D node distribution, a transparent mouse brain surface of an average anatomical standard mouse brain was rendered and used as anatomical reference.

### Statistical analysis

Data are presented as mean ± SEM. To check for general effects of temperature and genotype initially a two factor ANOVA was calculated with genotype and temperature range (either cold or heat) as fixed factors and the average BOLD parameter values (either activated volume or mean amplitude) per brain structure (n = 196) as dependent variables. For post hoc testing Student’s t-test (two-sample, n = 10 for WT and Na_V_1.8^−/−^) was used to calculate significant mean differences between genotypes and temperatures. Assumption for all performed Student’s t-tests: The subjects in each sample were randomly selected from a population, the sample data come from normally distributed populations of observation and the variances from the two samples were shown to be equal (therefore, homoscedastic Student’s t-test). Second-order group analysis on SPMs was performed using voxel-wise Student’s t-test. If appropriate, test statistics were corrected for multiple comparisons by FDR (q = 0.05, significance level α < 0.05). All data met the assumption of each statistical test applied.

## Results

We performed a second order group statistics of statistical parametric maps (SPMs) generated from the functional data set of Na_V_1.8^−/−^ and WT mice to visualize the spatial pattern of significantly different voxels throughout the brain upon cold stimulation (5 °C) (Fig. 1 (left column)).

Brain structures, which are important for processing of cold nociception, are the ones where the SPM values for Na_V_1.8^−/−^ are significantly different and smaller compared to WT (n = 10 for WT and Na_V_1.8^−/−^, respectively; for further calculation of significance see methods section). These structures encompassed subcortical brain structures including the zona incerta (ZI), the periaqueductal grey (PAG) and structures belonging to the thalamus (e.g. posterior thalamic nuclear group (thPo), ventral posteromedial thalamic nucleus (thVPM)) and the Hippocampus (Hip) (e.g. dorsal subiculum (hcSD), posterior dorsal hippocampus (hcPD), posterior layers of the dentate gyrus (hcDGp)). Other cortical structures like the retrosplenial cortex (cxRS), the cingulate cortex (cxCg), the parietal association cortex (cxPtA), parts of the somatosensory (cxSens) (primary somatosensory cortex (cxS1)) and of the motor cortex (primary and secondary motor cortex (M1/ M2)) also showed significantly larger SPM values in WT compared with Na_V_1.8^−/−^. In contrast, the only brain structures demonstrating higher SPM values in Na_V_1.8^−/−^ mice under cold stimulation were the amygdala (am) and the secondary somatosensory cortex (cxS2) (Fig. 1 (left column)).

In contrast to cold stimulation, the second order SPM group analysis of Na_V_1.8^−/−^ vs. WT mice upon heat stimulation at 55 °C revealed only few spatial regions which were found to be significantly different (Fig. 1 (right column)). In Na_V_1.8^−/−^ mice significantly stronger SPM values occurred only in M1, M2 and cxCg. In the WT significantly higher SPM values were found in a few subcortical structures like parts of the Hip (e.g. hcSD, anterior dorsal hippocampus (hcAD), hcDGp), the pretectal area (PTA), the ventromedial thalamic nucleus (thVM), the limbic output (outLimb) (e.g. medial hypothalamus (hyM), dorsomedial hypothalamus (hyDM), lateral hypothalamus (hyL), paraventricular hypothalamic nucleus (hyPV), ZI), and finally in the substantia nigra (SN) (n = 10 for WT and Na_V_1.8^−/−^, respectively; for further calculation of significance see methods section). Note the fact, that some structures showing only a single significant voxel (e.g. as for SN, hyM) depends on slice choice. Slices detected either before or behind the depicted one show also multiple significant voxels for these structures. Thus, the required minimum cluster size of 4 voxels defining activated brain volumes is also given for these structures, albeit this fact is not visible in one single slice presented.

First a two factor ANOVA was performed with genotype and temperature range (either cold or heat) as fixed factors and the average BOLD parameter values (either activated volume or mean amplitude) per brain structure (n = 196) as dependent variables. For both BOLD parameters ANOVA analysis of the cold stimulation revealed significant effects for genotype (activated volume: p = 1.22*10^−18^; F = 79.65; mean amplitude: p = 1.20*10^−10^, F = 42.04) and temperature (activated volume: p = 2.05*10^−7^; F = 11.43; mean amplitude: p = 4.57*10^−8^, F = 12.48) as well as significant interactions between the two factors (activated volume: p = 1.56*10^−11^; F = 18.08; mean amplitude: p = 1.54*10^−5^, F = 8.40). ANOVA analysis of mean BOLD amplitude due to heat stimulation also shows significant effects for both factors (genotype: p = 0.032, F = 4.61; temperature: p = 8.00*10^−41^, F = 67.12; interaction: p = 0.037, F = 2.83). However the ANOVA analysis of heat stimulation BOLD activated volume revealed a significant effect only for the temperature (p = 6.25*10^−23^, F = 36.72) and not for the genotype (p = 0.950; F = 0.004) without interactions (p = 0.731; F = 0.431). Subsequently, we performed the Student’s t-test as post hoc analysis to compare the total of activated volume and mean BOLD amplitude for cold and heat stimulation. The stimulus-response curves describing the total of activated volume, the mean BOLD amplitude as well as the mean activation probability for both genotypes and for cold and heat stimulation conditions, respectively, are given in (Fig. 2(a–f)).

For the cold experiment averaged brain responses revealed a lower total activated volume in Na_V_1.8^−/−^ compared with WT mice at all temperatures below 20 °C. Significant differences were noticed for 5 °C (p = 0.002; Student’s t-test, two-sample; α ≤ 0.01; n = 10 for WT and Na_V_1.8^−/−^, respectively) and 10 °C (p = 0.036; Student’s t-test, two-sample; α ≤ 0.05; n = 10 for WT and Na_V_1.8^−/−^, respectively), and showed a clear tendency for 0 °C (p = 0.056; Student’s t-test, two-sample; α ≤ 0.05; n = 10 for WT and Na_V_1.8^−/−^, respectively) (Fig. 2(a)). Also the mean BOLD amplitudes detected for Na_V_1.8^−/−^ mice were smaller at all temperatures below 20 °C compared with the WT (Fig. 2(b)). Additionally the mean activation probabilities were lower at all temperatures below 20 °C compared with the WT (Fig. 2(c)). Differences to WT being reduced to nearly 50% were found at 5 °C (Fig. 2(c)). Pure effects arising from the activation probability can be excluded.

Topical heat stimulation in contrast to cold stimulation revealed considerable less differences between Na_V_1.8^−/−^ and WT pertaining to all parameters (Fig. 2(d–f)). The only statistically significant difference upon heat stimulation was found for the mean BOLD amplitude at 55 °C (p = 0.037; Student’s t-test, two-sample; α ≤ 0.05; n = 10 for WT and Na_V_1.8^−/−^, respectively) (Fig. 2(e)).

**Figure 3:**
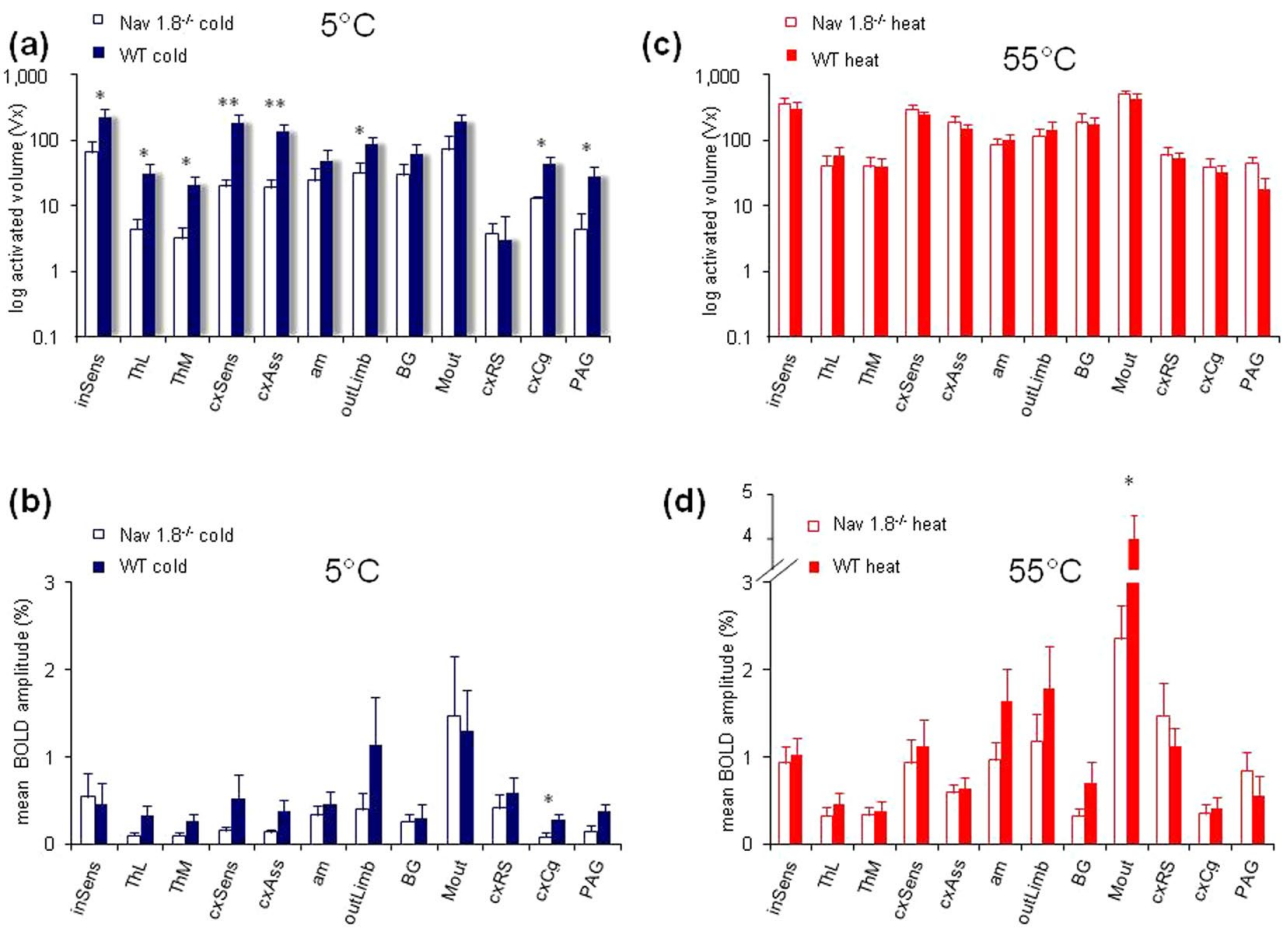
Comparison of activated volume and change of mean BOLD amplitude in functional groups of brain structures in Na_V_1.8^−/−^ and WT mice following cold (5 °C) and heat (55 °C) stimulation. A functional group analysis was calculated for two BOLD parameters (activated volume expressed as number of activated voxels: insets **a/c** (note the logarithmic scale) and mean BOLD amplitude: insets **b/d**) for cold stimulation (5 °C: blue) and heat stimulation (55 °C: red) separate for the two genotypes (Na_V_1.8^−/−^: open bars, WT: closed bars). Functional groups comprise brain structures with comparable functional/anatomical aspects. The functional group analysis provides a deeper insight, which functional systems show significant differences between Na_V_1.8^−/−^ and WT mice. The data are presented as mean values ± SEM, n = 10 for WT and Na_V_1.8^−/−^, respectively. Percentage declaration is referred to 100% of total mean BOLD amplitude. Significant differences between Na_V_1.8^−/−^ and WT mice for each stimulation condition are marked with asterisks: **p ≤ 0.01; *p ≤ 0.05; Student’s t-test, two-sample; uncorrected.

Furthermore, groups of brain structures sharing similar functions were compared regarding the parameters activated volume and mean BOLD amplitude for cold (5 °C) and heat (55 °C) stimulation separately for the two genotypes (Na_V_1.8^−/−^ and WT) (Fig. 3(a–d)).

As result for cold stimulation (5 °C), significantly reduced activated volumes were found between WT and Na_V_1.8^−/−^ by using in all following cases a Student’s t-test (two-sample; uncorrected; n = 10) in PAG (p = 0.048), in structures belonging to the sensory input (inSens) (p = 0.046), in the lateral and medial thalamus (ThL, ThM; p = 0.035, p = 0.029) and the outLimb (p = 0.048). Beyond, cortical structures like parts of the cxSens (p = 0.012), the association cortex (cxAss) (p = 0.007) and the cxCg (p = 0.019) showed markedly reduced activated volumes (Fig. 3(a)). Moreover, pertaining to the mean BOLD amplitude no significant difference was found at the level of functional groups between Na_V_1.8^−/−^ and WT at 5 °C except the cxCg, which solely showed a significantly reduced mean BOLD amplitude (p = 0.019) (Fig. 3(b)).

In contrast, comparing Na_V_1.8^−/−^ and WT upon heat stimulation conditions (55 °C), the functional group analysis revealed nearly identical activated volumes (Fig. 3(c)), and significantly lower mean BOLD amplitudes only in the motor output system (Mout) for the Na_V_1.8^−/−^ (p = 0.054) (Fig. 3(d)).

Subsequent single structure analysis, however, revealed several significant differences of the activated volume and mean BOLD amplitude in single brain structures for both stimulation conditions (5 °C and 55 °C) (see Supplementary Table S1). Again fewer and less pronounced significant differences were found upon heat compared to cold stimuli (for significance see Supplementary Table S1, respectively).

**Figure 4:**
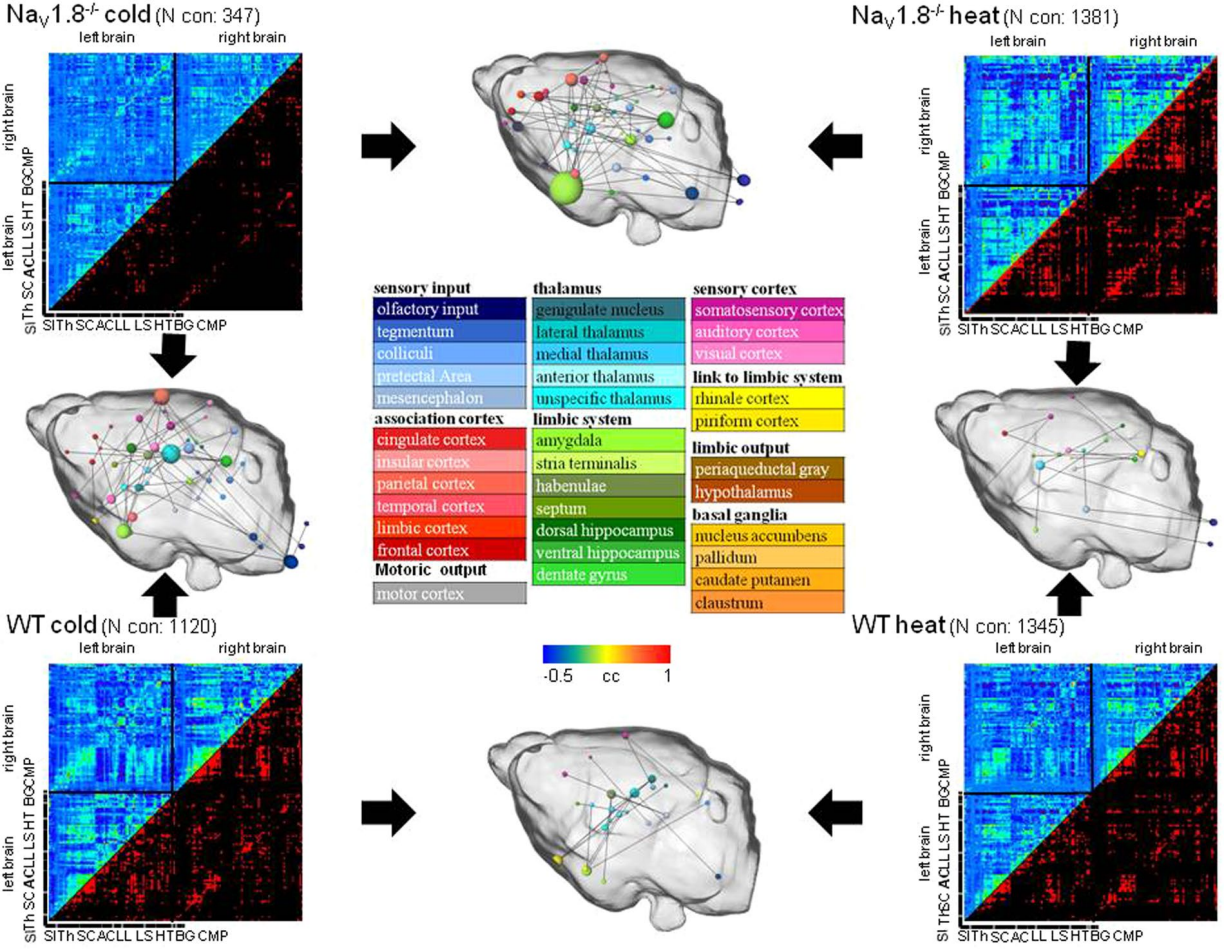
Changes in the interregional connectivity between pain-related brain structures in Na_V_1.8^−/−^ in contrast to WT mice for cold and heat stimulation conditions. Average correlation maps of time-courses of the different brain structures are given in the upper triangle, the corresponding FDR thresholded binary matrices are given in the respective lower triangle. A colour scale indicates the degree of correlation (blue, green, and red indicate little, medium and strong correlation, respectively). Upon cold stimulation conditions the signal spreads from the thalamus to higher-order pain structures in WT mice (green areas), whereas obviously weaker activity is found in Na_V_1.8^−/−^ mice (blue stripes). Correlation coefficients are given in the range from −0.5 (blue) to +1 (red). N = 10 for WT and Na_V_1.8^−/−^, respectively. Next, Student’s t-tests (two-sample; p ≤ 0.05, uncorrected) were calculated to reveal significant differences for the two genotypes and the two stimulation conditions and the result was transformed into 3D graphs consisting of nodes (brain structures, their 3D coordinates were derived from the Paxinos atlas^22^, color code see legend in the middle) and edges (connections, lines). The particular node size is given by the number of significantly different connections of a given Student’s t-test. Black arrows indicate the particular conditions which were tested. The graphs are rendered together with a transparent surface of the mouse brain. Abbreviations Fig. 4: SI, sensory input; Th, thalamus; SC, somatosensory cortex; AC, association cortex; LL, link to limbic system; LS, limbic system; HT, hypothalamus; BG, basal ganglia; C, cerebellum; M, motor cortex; P, periaqueductal gray.

Next, we investigated the functional connectivity by graph-theory between pain activated brain structures in order to get more insight in the changes of the dynamics of information flow within the whole brain network. The results derived from the connectivity network analysis of Na_V_1.8^−/−^ compared with WT mice are visualized in Fig. 4.

Cross-correlation matrices and significant correlations from the analysed brain structures for Na_V_1.8^−/−^ and WT mice upon cold or heat stimulation conditions are shown. The network analysis confirms that Na_V_1.8^−/−^ mice display dramatic alterations in the sensation of noxious cold stimulation compared to the WT, which is expressed in a considerable reduced interaction and obvious differences concerning the number of connections between pain brain structures upon cold nociception (number of connections: Nav1.8^−/−^: 347; WT: 1120). The 3D renderings in Fig. 4 show the brain structures with a significant larger node size (homoscedastic Student’s t-test, two-sample; α ≤ 0.05; n = 10 for WT and Na_V_1.8^−/−^) for a given comparison at their anatomical position within the mouse brain which is rendered as a transparent surface. Comparing networks for cold perception (i.e. Nav1.8^−/−^ cold/ WT cold) WT mice show a higher number of significantly different connections compared with Nav1.8^−/−^ mice, and therefore altered intraregional connectivity, included brain structures belonging to the thalamus (turquoise), the limbic system (Hippocampus, amygdala (green), the somatosensory (pink) and association cortex (red) as well as structures belonging to the sensory input (e.g. colliculi, PTA (blue)) (left side). Concerning heat stimulation (i.e. Nav1.8^−/−^ heat/ WT heat) Fig. 4 demonstrates that Na_V_1.8^−/−^ mice compared with WT show qualitatively quite the same functional connectivity pattern and consequently less prominent significantly different connections (number of connections: Nav1.8^−/−^: 1381; WT: 1345) and 3D rendering. Less but still significant effects were noticed in the limbic system (green), the thalamus (turquoise) and the sensory input (blue) (right side). The comparison of significant differences in connectivity upon cold and heat stimulation conditions for the Na_V_1.8^−/−^ group itself (i.e. Nav1.8^−/−^ cold/ Nav1.8^−/−^ heat) reveals significant differences concerning the number of significant connections especially in the limbic system (e.g. am, hippocampus (green)), the sensory input (blue), the sensory cortex (pink) and the association cortex (red), whereas the WT group for itself (i.e. WT cold/ WT heat) again showed less prominent differences.

## Discussion

By revealing significant changes of brain activation in Na_V_1.8^−/−^ and WT mice using BOLD fMRI upon noxious cold and heat stimulation conditions, we were able to detect certain brain structures as well as their functional connections, which especially underlay the influence of the voltage gated sodium channel Na_V_1.8. Thus, functionally, the two genotypes can be separated properly. BOLD fMRI has already been performed in rats^14, 23, 24^ and could recently be transferred to mice by our and other working groups^15, 18, 19^. Moreover, several experiments demonstrated the possibility of translating findings recorded in rodent pain imaging studies to humans^13, 14, 25^. Thus, we conclude, that our data on Nav1.8^−/−^ mice might also offer the opportunity to be translated to humans hinting at important facts about human cold pain processing and Nav1.8 as a potential pharmacological target.

In our experiments, the most resounding differences between WT and Na_V_1.8^−/−^ mice during cold stimulation (especially at 5 °C) impacted structures belonging to the inSens, the medial and lateral pain system, including thalamic regions and upstream many structures of the cxSens and cxAss, which are responsible for sensation, attention and integration of information. These findings demonstrate the importance of Na_V_1.8 concerning the function and interplay of these structures upon cold perception. Additionally, brainstem structures, responsible for descending pain modulation, like the PAG, which receives information from the nucleus accumbens (Acb) and is linked to the limbic coordination of fear and escape reactions^26, 27^, were affected. Thus, we conclude, that the lack of Na_V_1.8 might also modify descending pathways of the antinociceptive pain system.

Additionally, structures representing the affective motivational dimensions of pain were significantly affected by the Na_V_1.8 knockout, i.e. the ThM, the cxCg and the cxRS. Thus, nociception in mice seems to be a perceptual experience that depends also on cerebral processing and is also associated with changes in motivational behaviour^28^.

In human studies Tracey *et al*. also detected changes especially concerning the activated volume in thalamic nuclei, the basal ganglia (BG) and insula (cxIns) upon noxious cold (5 °C) and heat (46 °C) stimulation to the dorsum of the left hand of healthy male subjects, respectively. Cortical areas which were further activated during both stimulation conditions included the cxCg and the cxSens (cxS1 and cxS2 (weaker for noxious cold)), the caudate Putamen (CPu), the premotor and motor cortices as well as prefrontal and inferior parietal cortex^12^. Mohr *et al*. also detected the prefrontal cortex, the thalamus as well as the cxS1 as important brain structures involved in cold pain perception^29^. Moreover, Seifert *et al*. discovered that the anterior cxCg and the BG were highly impacted by cold allodynia^27^. In agreement to these human studies we detected significant differences in size and activation strength in exactly these structures (except insula) in the mouse brain of WT and Na_V_1.8^−/−^ mice in response to noxious cold stimulation as well, pointing to an essential role of Na_V_1.8 for the coordination and interplay of these structures and their essential function in cold pain processing. Tracey *et al*. observed that upon cold and heat stimulation most structures were activated bilaterally with a slight dominance for contralateral activation^12^. Further fMRI experiments also revealed a widespread bilateral brain activation in rodents^25^ and humans^26^ upon one-sided stimulation. The same phenomenon could be observed in our experiments on mice showing that although the stimulation was executed on the right hindpaw, no clear tendency towards any laterality of activation was obvious. Thus, with our data presented here, we were able to show the striking influence of Na_V_1.8 on cold associated pain states at the level of the CNS. The high comparability of our fMRI data with human data, mentioned above^12, 27, 29^, gives rise to a possible translation from mouse to humans allowing for presenting and functional validation of Nav1.8 as a potential target for the development of new drugs for cold associated pain states in humans like allodynia.

Furthermore, our results also fit well with the previously observed phenomenon that the loss of Na_V_1.8 resulted in a very distinct phenotype in the cold plate test, where genetically modified Na_V_1.8^−/−^ mice or mice, where Na_V_1.8 expressing neurons were killed by diphtheria toxin (DTA mice), show negligible responses to noxious cold temperatures as well as diminished responses to mechanical stimulation^1^.

In previous studies we were also able to show that Na_V_1.8 is essential for behavioural responses below 10 °C, especially at 5 °C and 0 °C^2^. Until now, this behaviour was explained by the phenomenon that the normal processes are interrupted and that the inactivation properties of Na_V_1.8 are largely cold-resistant, keeping the sensitivity for cold pain perception at a functional level^2^.

Our graph-theoretical connectivity analysis revealed significantly reduced interactions and connections between pain relevant structures at cold temperatures, pointing to an altered information flow within the whole brain network in Na_V_1.8^−/−^ mice starting with a reduced thalamic information transmission. This directly reflects the mentioned altered hyponociceptive phenotype of Na_V_1.8^−/−^ mice and linked electrophysiological features^2^.

Concerning heat stimulation conditions, Na_V_1.8^−/−^ mice presented slightly reduced activated brain volumes as well as reduced BOLD amplitudes linked with a similar but not identical activation pattern compared to WT. These results properly fit with the observations from the Hargreaves test described by Abrahamsen *et al*., who observed only a slight tendency towards an increased withdrawal latency at noxious thermal stimulation temperatures in DTA mice compared with the WT, i.e. a decrease in sensitivity to noxious heat stimulation^1^. On the other hand Akopian *et al*.^7^ and Abrahamsen *et al*.^1^ noticed a tendency towards a decreased withdrawal latency in the hot plate test at 50 °C and 55 °C stimulation temperature in Na_V_1.8^−/−^ or DTA mice compared with WT and a tendency towards an increased latency at 45 °C. This unsteady heat phenotype was reflected in our experiments by a reduction of the activated volume in Na_V_1.8^−/−^ mice at 45 °C and higher activated volumes at 50 °C and 55 °C (cf. Fig. 2(d)), which might explain the slightly altered sensitivity. With regard to our functional group analysis, we found a high response similarity in terms of activated volume (cf. Fig. 3(c)) and connectivity upon heat stimulation for Na_V_1.8^−/−^ mice and WT mice in general (cf. Fig. 4) - a result that goes along with the only slight but not significant behavioural differences between Na_V_1.8^−/−^ or DTA mice compared with WT upon heat stimulation in the hot plate and Hargreaves test described by Akopian *et al*.^7^ and by Abrahamsen *et al*.^1^. Consistent with these behavioural results our connectivity analysis showed drastic differences upon cold stimulation and only slight differences upon heat stimulation conditions. This shows that Na_V_1.8 is not only relevant for cold but also for heat pain processing, albeit to a quite lower extent.

Among numerous positive aspects fMRI in general bears also several limitations particular for animals. It is known, that effects of anaesthesia can influence pain perception. Many anaesthetics can function as analgesics and some even affect the neurovascular coupling or the BOLD signal acting as potent vasodilatators^19^. In rodent studies, isoflurane and α-Chloralose are the most commonly used anaesthetic agents^14^. α-Chloralose is expected to be toxic and therefore problematic for repetitive measurements. Moreover it leads to dominant cortical responses due to induction of hyperexcitability^14, 30^. Medetomidine, which is used in many fMRI rodent studies, offers depressant, analgetic, and musclerelaxant qualities. It has to be injected at a specific dose and is thus less controllable. Schroeter *et al*. noticed an increased latency of BOLD responses, i.e. the delay between stimulus onset and a positive ΔBOLD for medetomidine when time courses of ΔBOLD were extracted from S1HL upon electrical hindpaw stimulation in mice^31^, which is also a critical point for defining an exact signal peak for correct analysis. Moreover comparing the intra-individual and inter-individual variability of the BOLD fMRI scans Schroeter obtained better reproducibility for isoflurane (and propofol) anesthetized mice as compared to medetomidine and urethane, although the low correlation coefficients obtained for medetomidine and urethane may be largely attributed to the smaller response and should therefore be not over-interpreted^31^. Schroeter *et al*. also stated that nevertheless, for medetomidine, difficulties in obtaining reproducible responses from repetitive measurements in the same imaging session have been reported previously^18, 31, 32^. The relatively strong analgesic component of medetomidine was further in conflict with our experiments, studying noxious cold and heat signaling. An fMRI study by Bosshard *et al*. in mice demonstrated the feasibility of a robust BOLD fMRI protocol to study nociceptive processing in isoflurane anesthetized mice^33^. The authors claimed a high reliability of the isoflurane anaesthesia, which allows for detailed analysis of the temporal BOLD profile and for investigation of somatosensory and noxious signal processing in the brain^33^.

One further limitation due to the anaesthetic used is that data with low motion can only be acquired if the animal stays in a fixed position inside the scanner. Thus, anaesthesia has to be deep enough to suppress movement, but light enough to pass activation signaling, which requires an elaborated anaesthesia protocol. Thus, in our experiments we used isoflurane which is only slightly analgesic, allows easy administration, controlled dosing and produces highly reproducible BOLD signals^15, 19, 21, 34^.

Further limitations for fMRI scanning are that fMRI BOLD signals have a low time resolution compared to neuronal activity. Moreover, because BOLD signals measure indirect hemodynamic changes the underlying neural activity can not directly be measured and BOLD signals do not allow to differentiate excitatory and inhibitory processes. Additionally, external influences like scanner noise, might interfere. However, these influences remain constant during the whole experiment and also across different subjects. Conventional GLM analysis of the MRI time series ensures to capture real activation besides such confounders, which can be modelled accordingly in the GLM.

Na_V_1.8 is expressed only in peripheral neurons and is limited to sensory and myenteric neurons including small and medium-sized DRG neurons and their axons^3–5^. With our experiments we demonstrate, that besides the peripheral effect of Na_V_1.8, higher order processes including supraspinal/central mechanisms are involved altering the CNS processing of noxious cold stimuli and lead to changed interconnections in the CNS of Na_V_1.8^−/−^ mice. Thus, our results add altered profound CNS differences to the previous findings from electrophysiological and behavioural studies on Na_V_1.8.

We assume, that the altered phenotype of Na_V_1.8^−/−^ mice and the fact that Na_V_1.8^−/−^ mice do not perceive nociceptive aspects of strong cooling in contrast to their WT littermates is not only an expression of a just peripheral phenomenon going along with diminished peripheral transmission, but is also an effect of the upstream processing with reduced afferent input leading to altered subsequent nociceptive processing in the CNS and consequently altered connectivity between pain-relevant brain structures combined with a reorganization of the response to cold noxious stimuli.

Thus, with our present study we were able to show the impact of Na_V_1.8 on noxious cold (and to a less extent heat) signaling. Na_V_1.8 therefore represents an interesting target for the development of new analgesics for the treatment of especially cold pain states like cold allodynia. Along with our study, we assume, that such drugs might not only act in the periphery, where Na_V_1.8 is mainly located, but also in the central nervous system effectively diminishing cold pain in associated pain states and could thus offer a advantageous new therapeutic approach.

## Electronic supplementary material

Supplementary information
