# Supplementary material for: Activity and connectivity changes of central projection areas revealed by functional magnetic resonance imaging in NaV1.8-deficient mice upon cold signaling
Source: Sci Rep. 2017 Apr 3;7:543. doi: 10.1038/s41598-017-00524-x (PMC5428718; doi:10.1038/s41598-017-00524-x)
Supplement: Supplementary file 1 — Supplementary information [file 41598_2017_524_MOESM1_ESM.pdf]

Title: Activity and connectivity changes of central projection areas revealed by functional magnetic resonance imaging in $Na_V1.8$-deficient mice upon cold signaling

Authors and author addresses:

Heindl-Erdmann C[1], Zimmermann K[2], Reeh P[3], Brune K[1], Hess A[1*]

[1] Friedrich-Alexander University Erlangen-Nürnberg (FAU), Institute of Experimental and Clinical Pharmacology and Toxicology, Fahrstraße 17, 91054 Erlangen, Germany

[2] Friedrich-Alexander University Erlangen-Nürnberg (FAU), Department of Anaesthesia, University Hospital Erlangen, Krankenhausstraße 12, 91054 Erlangen, Germany

[3] Friedrich-Alexander University Erlangen-Nürnberg (FAU), Institute of Physiology und Pathophysiology, Universitätsstraße 17, 91054 Erlangen, Germany

[*] Corresponding author: A. Hess, Institute of Experimental and Clinical Pharmacology and Toxicology, Fahrstrasse 17, 91054 Erlangen, Germany

E-mail: andreas.hess@fau.de

telephone number: +49-9131-85-22003

fax number: +49-9131-85-22774

Supplementary Information

Table S1

	5°C				55°C			
	activated volume		BOLD amplitude		activated volume		BOLD amplitude	
	$Na_v1.8^{-/-}$	WT	$Na_v1.8^{-/-}$	WT	$Na_v1.8^{-/-}$	WT	$Na_v1.8^{-/-}$	WT
TN r	0.00 ± 0.00	1.50 ± 0.79	0.00 ± 0.00	0.56 ± 0.29	1.00 ± 0.45	2.00 ± 0.91	0.66 ± 0.37	0.70 ± 0.33
TN l	0.00 ± 0.00	1.50 ± 0.37	0.00 ± 0.00	0.19 ± 0.09	2.13 ± 0.60	3.38 ± 1.50	0.91 ± 0.26	0.37 ± 0.17
VTA r	0.50 ± 0.24	1.75 ± 0.94	0.24 ± 0.11	0.23 ± 0.11	2.00 ± 0.96	1.00 ± 0.89	0.46 ± 0.21	0.19 ± 0.17
VTA l	0.13 ± 0.11	3.25 ± 1.17	0.00 ± 0.00	0.35 ± 0.14	5.13 ± 2.09	2.50 ± 0.99	0.44 ± 0.17	0.29 ± 0.14
Red r	0.13 ± 011	2.50 ± 1.63	0.00 ± 0.00	0.23 ± 0.10	2.88 ± 1.37	0.75 ± 0.55	0.41 ± 0.15	0.20 ± 0.15
Red l	0.00 ± 0.00	3.38 ± 1.33	0.00 ± 0.00	0.33 ± 0.10	5.63 ± 1.97	1.75 ± 0.73	0.47 ± 0.17	0.21 ± 0.14
IP	0.13 ± 0.11	1.75 ± 0.79	0.10 ± 0.08	0.27 ± 0.13	2.00 ± 0.93	0.25 ± 0.22	0.75 ± 0.23	0.18 ± 0.16
IC r	0.00 ± 0.00	16.00 ± 4.14	0.00 ± 0.00	0.58 ± 0.17	28.88 ± 7.77	22.50 ± 4.47	2.88 ± 0.46	1.44 ± 0.40
IC l	0.50 ± 0.45	7.50 ± 2.31	0.00 ± 0.00	0.25 ± 0.13	63.50 ± 8.58	22.13 ± 7.31	2.31 ± 0.26	1.80 ± 0.49
PTA r	0.00 ± 0.00	3.88 ± 1.55	0.00 ± 0.00	0.09 ± 0.05	2.63 ± 1.30	5.38 ± 3.22	0.20 ± 0.11	0.40 ± 0.19
PTA l	0.13 ± 1.11	3.75 ± 0.11	0.00 ± 1.32	0.18 ± 0.06	9.63 ± 3.35	4.50 ± 1.82	0.45 ± 0.13	0.42 ± 0.13
thLP r	0.75 ± 0.37	2.88 ± 1.22	0.15 ± 0.07	0.17 ± 0.06	2.63 ± 0.96	3.75 ± 1.50	0.22 ± 0.09	0.44 ± 0.17
thLP l	0.00 ± 0.00	1.75 ± 0.84	0.00 ± 0.00	0.07 ± 0.05	6.00 ± 2.34	3.38 ± 1.41	0.17 ± 0.08	0.23 ± 0.09
thPo r	0.25 ± 0.15	1.75 ± 0.82	0.10 ± 0.09	0.23 ± 0.07	2.00 ± 0.99	3.25 ± 1.71	0.22 ± 0.10	0.27 ± 0.18
thPo l	0.25 ± 0.22	4.25 ± 1.44	0.10 ± 0.09	0.29 ± 0.09	5.75 ± 2.71	4.25 ± 1.63	0.30 ± 0.10	0.29 ± 0.11
thVM r	0.00 ± 0.00	1.63 ± 0.61	0.00 ± 0.00	0.29 ± 0.11	1.13 ± 0.89	4.75 ± 2.23	0.17 ± 0.12	0.32 ± 0.15
thVM l	0.38 ± 0.34	2.00 ± 1.00	0.05 ± 0.04	0.24 ± 0.14	1.00 ± 0.51	4.50 ± 1.74	0.23 ± 0.13	0.61 ± 0.21
thVL r	0.38 ± 0.35	1.00 ± 0.77	0.04 ± 0.04	0.16 ± 0.10	3.50 ± 2.00	3.63 ± 2.10	0.14 ± 0.07	0.35 ± 0.14
thVL l	0.00 ± 0.00	2.63 ± 1.45	0.00 ± 0.00	0.25 ± 0.12	4.88 ± 2.40	7.88 ± 2.55	0.28 ± 0.11	0.46 ± 0.12
thVPM r	0.00 ± 0.00	2.25 ± 1.04	0.00 ± 0.00	0.20 ± 0.08	2.25 ± 1.25	2.25 ± 1.19	0.11 ± 0.06	0.22 ± 0.10
thVPM l	0.50 ± 0.45	6.88 ± 2.54	0.05 ± 0.05	0.32 ± 0.10	4.88 ± 1.78	8.00 ± 2.35	0.19 ± 0.07	0.29 ± 0.10
thVPL r	0.00 ± 0.00	0.38 ± 0.24	0.00 ± 0.00	0.12 ± 0.07	1.38 ± 0.61	2.38 ± 0.88	0.12 ± 0.06	0.34 ± 0.14
thVPL l	0.00 ± 0.00	1.50 ± 0.59	0.00 ± 0.00	0.16 ± 0.07	1.13 ± 0.52	4.00 ± 0.93	0.23 ± 0.10	0.41 ± 0.13

thMD r	1.00 ± 0.77	4.50 ± 1.60	0.05 ± 0.05	0.21 ± 0.09	10.63 ± 5.99	10.25 ± 4.50	0.29 ± 0.11	0.33 ± 0.15
thMD l	0.00 ± 0.00	9.50 ± 3.03	0.00 ± 0.00	0.32 ± 0.06	14.38 ± 4.56	11.50 ± 4.62	0.44 ± 0.10	0.21 ± 0.10
thLD r	0.00 ± 0.00	0.63 ± 0.24	0.00 ± 0.00	0.25 ± 0.09	3.00 ± 1.16	3.00 ± 1.18	0.15 ± 0.07	0.34 ± 0.13
thLD l	0.00 ± 0.00	1.38 ± 0.45	0.00 ± 0.00	0.16 ± 0.04	2.63 ± 1.09	2.63 ± 1.11	0.18 ± 0.10	0.24 ± 0.09
thSM r	0.00 ± 0.00	0.25 ± 0.22	0.00 ± 0.00	0.05 ± 0.05	1.25 ± 0.81	1.25 ± 0.58	0.21 ± 0.13	0.40 ± 0.19
thSM l	0.00 ± 0.00	0.63 ± 0.38	0.00 ± 0.00	0.10 ± 0.06	0.75 ± 0.44	1.63 ± 0.86	0.11 ± 0.06	0.24 ± 0.11
cxPtA r	0.13 ± 0.11	5.25 ± 1.64	0.00 ± 0.00	0.20 ± 0.08	2.38 ± 1.41	2.00 ± 0.72	0.22 ± 0.13	0.82 ± 0.29
cxPtA l	0.00 ± 0.00	7.88 ± 2.00	0.00 ± 0.00	0.30 ± 0.09	1.88 ± 1.20	0.88 ± 0.57	0.23 ± 0.11	0.24 ± 0.15
cxRS r	0.5 ± 0.24	25.63 ± 5.61	0.11 ± 0.07	0.44 ± 0.10	26.50 ± 9.00	22.38 ± 3.25	1.66 ± 0.46	1.63 ± 0.30
cxRS l	1.25 ± 0.28	26.75 ± 6.26	0.60 ± 0.24	0.40 ± 0.09	24.00 ± 5.07	30.38 ± 3.98	1.01 ± 0.23	0.66 ± 0.15
cxCg r	0.38 ± 0.34	5.25 ± 52.06	0.00 ± 0.00	0.14 ± 0.05	13.63 ± 5.14	13.75 ± 3.94	0.35 ± 0.11	0.28 ± 0.08
cxCg l	1.50 ± 0.72	5.25 ± 2.36	0.08 ± 0.04	0.35 ± 0.08	20.50 ± 7.22	14.88 ± 3.66	0.35 ± 0.11	0.29 ± 0.08
cxPrL r	0.00 ± 0.00	0.63 ± 0.38	0.00 ± 0.00	0.26 ± 0.16	2.25 ± 1.01	1.25 ± 0.89	0.55 ± 0.15	0.07 ± 0.06
cxPrL l	0.00 ± 0.00	1.25 ± 0.53	0.00 ± 0.00	0.40 ± 0.11	3.38 ± 1.36	0.88 ± 0.43	0.66 ± 0.17	0.15 ± 0.09
cxPdD r	0.00 ± 0.00	0.88 ± 0.78	0.00 ± 0.00	0.16 ± 0.15	0.00 ± 0.00	0.50 ± 0.29	0.00 ± 0.00	0.01 ± 0.00
cxPdD l	0.00 ± 0.00	0.38 ± 0.34	0.00 ± 0.00	0.18 ± 0.17	0.13 ± 0.11	0.38 ± 0.33	0.00 ± 0.00	0.11 ± 0.10
cxOrb r	0.00 ± 0.00	0.88 ± 0.43	0.00 ± 0.00	0.034 ± 0.03	0.50 ± 0.34	2.38 ± 0.89	0.10 ± 0.07	0.24 ± 0.11
cxOrb l	0.13 ± 0.11	0.63 ± 0.56	0.00 ± 0.00	0.00 ± 0.00	0.00 ± 0.00	0.63 ± 0.56	0.00 ± 0.00	0.00 ± 0.00
cxIns r	4.38 ± 1.64	13.88 ± 4.71	0.29 ± 0.11	0.42 ± 0.15	29.75 ± 4.24	20.50 ± 4.19	0.89 ± 0.18	1.12 ± 0.25
cxIns l	1.38 ± 0.84	4.50 ± 1.47	0.16 ± 0.09	0.22 ± 0.08	15.25 ± 5.52	8.50 ± 2.21	0.58 ± 0.19	0.49 ± 0.24
cxEnt r	3.00 ± 1.00	19.13 ± 6.00	0.67 ± 0.19	0.38 ± 0.14	31.00 ± 3.89	51.75 ± 14.63	1.84 ± 0.34	1.34 ± 0.29
cxEnt l	1.50 ± 0.53	11.38 ± 3.62	0.45 ± 0.20	0.58 ± 0.17	42.13 ± 9.47	47.00 ± 11.32	1.77 ± 0.35	2.13 ± 0.57
cxS1BF r	1.75 ± 0.91	10.38 ± 2.72	0.12 ± 0.06	0.36 ± 0.08	14.88 ± 5.64	21.38 ± 4.83	0.46 ± 0.18	1.59 ± 0.42
cxS1BF l	0.25 ± 0.22	2.38 ± 0.91	0.00 ± 0.00	0.22 ± 0.10	22.50 ± 8.72	20.88 ± 5.74	0.49 ± 0.15	0.38 ± 0.12
cxS1r r	1.13 ± 0.43	1.88 ± 1.01	0.31 ± 0.12	0.11 ± 0.06	20.63 ± 3.44	7.38 ± 2.93	1.23 ± 0.23	1.28 ± 0.46
cxS1r l	0.00 ± 0.00	1.63 ± 0.88	0.00 ± 0.00	0.03 ± 0.03	9.63 ± 3.88	6.00 ± 2.49	1.04 ± 0.39	0.36 ± 0.16
cxS2 r	0.25 ± 0.15	9.375 ± 3.03	0.05 ± 0.05	0.30 ± 0.11	19.75 ± 3.48	12.38 ± 3.07	0.82 ± 0.11	1.62 ± 0.57
cxS2 l	0.75 ± 0.55	0.25 ± 0.22	0.16 ± 0.10	0.05 ± 0.05	7.25 ± 4.17	5.13 ± 2.09	0.18 ± 0.08	0.59 ± 0.34

hcAD r	1.25 ± 0.500	7.63 ± 3.87	0.15 ± 0.05	0.23 ± 0.05	17.63 ± 7.48	23.25 ± 8.21	0.33 ± 0.08	0.43 ± 0.09
hcAD l	0.38 ± 0.33	14.63 ± 5.79	0.05 ± 0.04	0.18 ± 0.03	18.25 ± 6.14	26.75 ± 7.20	0.26 ± 0.09	0.40 ± 0.09
hcPD r	2.25 ± 1.23	23.63 ± 7.62	0.21 ± 0.10	0.27 ± 0.06	20.13±11.54	13.50 ± 4.38	0.54 ± 0.16	0.64 ± 0.20
hcPD l	4.38 ± 1.80	15.00 ± 4.70	0.10 ± 0.06	0.16 ± 0.04	33.00±16.59	41.00 ± 10.96	0.37 ± 0.13	0.84 ± 0.15
hcSD r	0.25 ± 0.22	6.88 ± 2.38	0.00 ± 0.00	0.27 ± 0.09	4.75 ± 2.13	4.38 ± 1.70	0.27 ± 0.13	0.85 ± 0.38
hcSD l	0.38 ± 0.34	5.63 ± 2.19	0.05 ± 0.04	0.20 ± 0.07	9.50 ± 4.65	11.00 ± 3.12	0.17 ± 0.11	0.59 ± 0.17
hcV r	4.25 ± 1.95	5.50 ± 1.88	0.21 ± 0.08	0.18 ± 0.07	7.50 ± 4.14	19.13 ± 8.33	0.23 ± 0.13	0.40 ± 0.18
hcV l	5.25 ± 2.90	6.25 ± 2.61	0.19 ± 0.09	0.50 ± 0.13	30.75 ± 12.72	36.88 ± 10.20	0.88 ± 0.21	0.63 ± 0.16
hcSV r	0.63 ± 0.56	1.50 ± 0.44	0.00 ± 0.00	0.16 ± 0.10	2.00 ± 1.06	7.38 ± 2.15	0.03 ± 0.02	0.58 ± 0.20
hcSV l	0.38 ± 0.24	0.75 ± 0.33	0.04 ± 0.03	0.35 ± 0.17	1.88 ± 1.20	3.88 ± 1.16	0.87 ± 0.48	0.73 ± 0.22
hcDGp r	1.5 ± 0.79	19.75 ± 6.73	0.32 ± 0.14	0.39 ± 0.12	12.88 ± 7.44	16.75 ± 5.27	0.28 ± 0.10	0.59 ± 0.14
hcDGp l	5.38 ± 2.71	13.50 ± 3.62	0.23 ± 0.11	0.36 ± 0.14	31.63 ± 12.49	35.88 ± 7.01	0.25 ± 0.10	0.62 ± 0.09
amM r	0.75 ± 0.44	1.38 ± 0.72	0.28 ± 0.16	0.22 ± 0.14	0.50 ± 0.45	6.00 ± 1.69	0.40 ± 0.36	1.21 ± 0.32
amM l	0.50 ± 0.34	2.75 ± 1.38	0.33 ± 0.21	0.40 ± 0.18	7.63 ± 1.66	8.25 ± 2.12	1.32 ± 0.41	2.72 ± 0.77
amCo r	1.13 ± 0.36	3.63 ± 0.75	0.60 ± 0.19	1.21 ± 0.24	7.75 ± 2.46	8.75 ± 1.61	1.44 ± 0.38	2.32 ± 0.38
amCo l	0.63 ± 0.45	2.63 ± 1.16	0.27 ± 0.24	0.93 ± 0.39	4.88 ± 1.64	5.75 ± 1.45	1.10 ± 0.26	2.49 ± 0.79
amBM r	0.50 ± 0.24	0.88 ± 0.52	0.32 ± 0.21	0.09 ± 0.08	1.63 ± 0.88	5.75 ± 1.65	1.39 ± 0.63	2.46 ± 0.77
amBM l	0.88 ± 0.66	2.75 ± 1.11	0.39 ± 0.23	1.03 ± 0.43	10.25 ± 2.22	6.25 ± 1.98	1.15 ± 0.26	1.29 ± 0.34
amBL r	2.50 ± 1.49	0.75 ± 0.47	0.23 ± 0.10	0.10 ± 0.06	6.50 ± 1.78	10.75 ± 2.83	1.15 ± 0.40	1.85 ± 0.55
amBL l	0.88 ± 0.66	9.50 ± 3.08	0.19 ± 0.13	0.52 ± 0.17	6.5 ± 1.78	10.75 ± 2.83	1.15 ± 0.63	1.85 ± 0.77
hyM r	0.88 ± 0.66	3.00 ± 0.96	0.37 ± 0.22	2.23 ± 1.21	1.38 ± 0.56	7.38 ± 1.80	1.69 ± 0.95	5.50 ± 1.59
hyM l	1.38 ± 1.11	2.38 ± 0.74	0.36 ± 0.21	4.59 ± 1.76	1.25 ± 0.65	11.50 ± 2.38	2.38 ± 1.06	4.76 ± 1.17
hyL r	2.00 ± 1.42	6.25 ± 1.80	0.66 ± 0.30	1.10 ± 0.34	8.00 ± 3.57	17.38 ± 6.39	1.99 ± 0.67	1.37 ± 0.28
hyL l	2.00 ± 1.35	7.25 ± 3.08	0.35 ± 0.21	2.03 ± 0.70	8.75 ± 3.30	25.25 ± 8.48	2.35 ± 0.93	2.59 ± 0.62
hyArc r	0.00 ± 0.00	0.25 ± 0.15	0.00 ± 0.00	1.90 ± 1.35	1.13 ± 0.39	0.38 ± 0.24	2.43 ± 1.18	1.76 ± 1.04
hyArc l	0.00 ± 0.00	0.88 ± 0.39	0.00 ± 0.00	3.65 ± 1.93	1.25 ± 0.65	0.38 ± 0.16	1.89 ± 1.13	3.05 ± 1.39
hyPV r	0.25 ± 0.22	0.13 ± 0.11	0.18 ± 0.16	0.07 ± 0.07	0.13 ± 0.11	1.38 ± 0.72	0.15 ± 0.13	0.43 ± 0.22
hyPV l	0.38 ± 0.34	0.00 ± 0.00	0.20 ± 0.18	0.00 ± 0.00	0.00 ± 0.00	2.88 ± 1.14	0.00 ± 0.00	2.25 ± 1.25

hyDM r	0.00 ± 0.00	0.50 ± 0.45	0.00 ± 0.00	0.13 ± 0.12	0.38 ± 0.16	2.50 ± 1.24	2.52 ± 1.78	0.45 ± 0.22
hyDM l	0.00 ± 0.00	0.38 ± 0.24	0.00 ± 0.00	0.30 ± 0.18	0.13 ± 0.11	1.50 ± 1.34	2.20 ± 1.97	0.14 ± 0.12
hyPo r	0.25 ± 0.22	1.25 ± 0.69	0.05 ± 0.05	0.13 ± 0.07	0.63 ± 0.56	2.88 ± 1.56	0.13 ± 0.12	0.47 ± 0.24
hyPo l	0.00 ± 0.00	1.38 ± 0.79	0.00 ± 0.00	0.18 ± 0.10	0.88 ± 0.78	2.13 ± 1.16	0.00 ± 0.00	0.52 ± 0.25
ZI r	0.25 ± 0.22	4.38 ± 1.37	0.00 ± 0.00	0.38 ± 0.10	3.50 ± 2.16	5.63 ± 2.84	0.24 ± 0.14	0.42 ± 0.21
ZI l	0.38 ± 0.24	3.25 ± 1.25	0.10 ± 0.09	0.21 ± 0.11	2.75 ± 1.56	9.63 ± 2.54	0.26 ± 0.12	0.55 ± 0.16
PAG	4.38 ± 3.30	28.38 ± 10.82	0.15 ± 0.08	0.37 ± 0.09	44.88 ± 11.95	18.13 ± 8.25	0.84 ± 0.22	0.55 ± 0.23
CoM	0.50 ± 0.45	2.25 ± 1.36	0.07 ± 0.06	0.32 ± 0.28	13.13 ± 3.79	1.13 ± 0.60	1.50 ± 0.62	0.75 ± 0.38
Cpu r	1.63 ± 0.77	18.63 ± 5.60	0.10 ± 0.06	0.35 ± 0.13	53.00 ± 24.53	66.63 ± 26.53	0.34 ± 0.08	0.43 ± 0.10
Cpu l	7.63 ± 3.91	9.50 ± 3.44	0.22 ± 0.08	0.28 ± 0.08	58.50 ± 22.76	37.88 ± 11.29	0.30 ± 0.09	0.35 ± 0.12
GPL r	0.25 ± 0.15	0.25 ± 0.15	0.05 ± 0.04	0.09 ± 0.07	5.25 ± 3.26	5.50 ± 3.31	0.15 ± 0.08	0.43 ± 0.25
GPL l	0.38 ± 0.24	1.38 ± 0.58	0.06 ± 0.05	0.19 ± 0.11	6.00 ± 3.48	5.75 ± 2.05	0.18 ± 0.10	0.49 ± 0.10
Cl r	0.00 ± 0.00	0.88 ± 0.31	0.00 ± 0.00	0.10 ± 0.04	2.25 ± 0.77	2.38 ± 0.65	0.21 ± 0.08	0.72 ± 0.20
Cl l	0.25 ± 0.15	0.75 ± 0.33	0.06 ± 0.05	0.34 ± 0.18	2.38 ± 1.28	1.38 ± 0.65	0.18 ± 0.08	0.23 ± 0.14
M2 r	2.50 ± 1.66	14.00 ± 4.30	0.09 ± 0.08	0.30 ± 0.11	49.25 ± 11.17	22.88 ± 5.22	0.96 ± 0.17	1.10 ± 0.16
M2 l	3.13 ± 2.10	22.25 ± 6.60	0.17 ± 0.10	0.37 ± 0.13	40.25 ± 6.42	22.50 ± 4.67	0.81 ± 0.12	0.85 ± 0.19
M1 r	2.25 ± 0.92	6.63 ± 2.29	0.16 ± 0.07	0.77 ± 0.18	35.38 ± 6.90	19.13 ± 6.77	0.97 ± 0.21	1.27 ± 0.25
M1 l	1.00 ± 0.51	14.13 ± 4.21	0.22 ± 0.10	0.20 ± 0.10	33.25 ± 5.61	21.63 ± 4.79	1.08 ± 0.21	1.15 ± 0.27

Table S1: Quantification at single structure level: activated volume and mean BOLD signal amplitude for $Na_V1.8^{-/-}$ and WT mice at 5 °C and 55 °C.

In addition to the functional group analysis (**Figure 3**) the single structures were statistically compared between $Na_V1.8^{-/-}$ and WT mice, giving detailed information about significant differences at the level of single brain structures.

In the brainstem most striking differences occurred in the periaqueductal gray (PAG), in the midbrain in the pretectal area (PTA), and -referring to the lateral pain system- in some nuclei of the thalamus, especially the ventromedial thalamic nucleus (thVM), the ventral

posteromedial thalamic nucleus (thVPM), the ventral posterolateral thalamic nucleus (thVPL) and the posterior thalamic nuclear group (thPo) as well as in the lateral posterior thalamic nucleus (thLP). Beyond, parts of the somatosensory cortex (cxSens) (e.g. secondary somatosensory cortex (cxS2) and primary sensory cortex barrel field (cxS1BF)), parts of the association cortex (cxAss) (e.g. retrosplenial cortex (cxRS), the parietal association cortex (cxPtA), parts of the frontal association cortex (orbital cortex (cxOrb)) and the prelimbic cortex (cxPrL)) and structures forming part of the sensory input (inSens) (e.g. the ventral tegmental area (VTA), the red nucleus (Red) and the inferior colliculus (IC) showed markedly reduced activated volumes.

Components of the medial pain system, like parts of the medial thalamus (ThM) (e.g. the laterodorsal thalamic nucleus (thLD) as well as the mediodorsal thalamus (thMD)) and parts of the cxAss (e.g. cingulate cortex (cxCg)) showed significantly lower activated brain volumes as well. This applies also to the limbic system including the entorhinal cortex (cxEnt), sub-structures of the hippocampus (Hip) (e.g. anterior dorsal hippocampus (hcAD) and posterior dorsal hippocampus (hcPD), the dorsal subiculum (hcSD) and posterior layers of the dentate gyrus (hcDGp)), parts of the amygdala (am) (e.g. the basolateral amygdaloid nucleus (amBL) and the cortical amygdala (amCo)) and limbic output (outLimb) structures like arcuate hypothalamic nucleus (hyArc) as well as zona incerta (ZI). Of note, no significant differences were recognized in the insular cortex (cxIns).

Moreover single structures of the motoric output (Mout) (i.e. primary motor cortex (M1) and secondary motor cortex (M2)) and structures of the basalganglia (BG) (e.g. caudate putamen (CPu) and Claustrum (Cl)) showed also significantly reduced activated volumes in $Na_V1.8^{-/-}$ mice.

Concerning the mean BOLD signal amplitude $Na_V1.8^{-/-}$ mice showed significantly lower mean BOLD signal amplitudes at 5 °C in some thalamic nuclei (e.g. in the ventrolateral thalamic nucleus (thVL), thVM, thVPM, thVPL and the thLD as well as the thMD), in inSens

structures like the tegmental nuclei (TN), the VTA, the Red, the IC as well as the PTA, in parts of the cxSens (e.g. cxS2, cxS1BF), the M1 and parts of the cxAss (e.g. cxPtA, cxRS, cxCg, cxPrL), the medial and lateral hypothalamus (HT) (e.g. medial hypothalamus (hyM) and lateral hypothalamus (hyL)) and parts of the Hip (e.g. hcAD and hcSD). Also parts of the BG including the Cl as well as of the outLimb (e.g. ZI) showed significantly reduced mean BOLD signal amplitudes. No significant differences were observed in the PAG, the thLP, the thPo, the hcPD and the hcDGp, cxEnt, parts of the am (e.g. amBL, amCo), the hyArc, the CPu, the M2 and the cxIns.

Upon heat stimulation, in contrast to cold stimulation, much less significant differences for both BOLD parameters were found. Regarding the activated volume no structure of the ThM and only the thVPL in the lateral thalamus (ThL) showed a significant reduction. Further significant reductions were found in the hyM and the paraventricular hypothalamic nucleus (hyPV), the medial amygdaloid nucleus (amM), the basomedial amygdaloid nucleus (amBM), the amBL, in the Hip (e.g. ventral subiculum (hcSV)) as well as in ZI. As already shown in **Fig. 1**, a significantly higher activated volume was observed in $Na_V1.8^{-/-}$ mice in the motor (M2) and parts of the cxSens (e.g. primary somatosensory cortex (cxS1)) as well as in inSens structures (e.g. IC) and outLimb structures (e.g. corpora mammillaria (CoM)). Markedly, no significant differences were detected in the cxAss, as well as in the link to the limbic system.

Significantly lower mean BOLD amplitudes in $Na_V1.8^{-/-}$ mice were noticed in structures belonging to the somatosensory cortex (e.g. cxS1BF) and parts of the Hip (e.g. hcPD, hcSD, hcSV and hcDGp), the posterior hypothalamus (hyPo), the am (e.g. amBL) and the BG (e.g. in the lateral globus pallidus (GPL) and Cl). Significantly higher mean BOLD amplitudes in $Na_V1.8^{-/-}$ were only observed for IC as well as in the cxPrL. Interestingly, again no significant differences in the mean BOLD amplitude in reaction to noxious heat stimulation were observed in all thalamic brain structures analyzed.

Data are presented as mean values ± SEM, n=10. Significant differences (uncorrected) between $Na_V1.8^{-/-}$ and WT are highlighted in dark gray ($p \leq 0.001$), middle gray ($p \leq 0.01$) and light gray ($p \leq 0.05$); two-sample Student´s t-test, n=10, respectively.

Figure S1

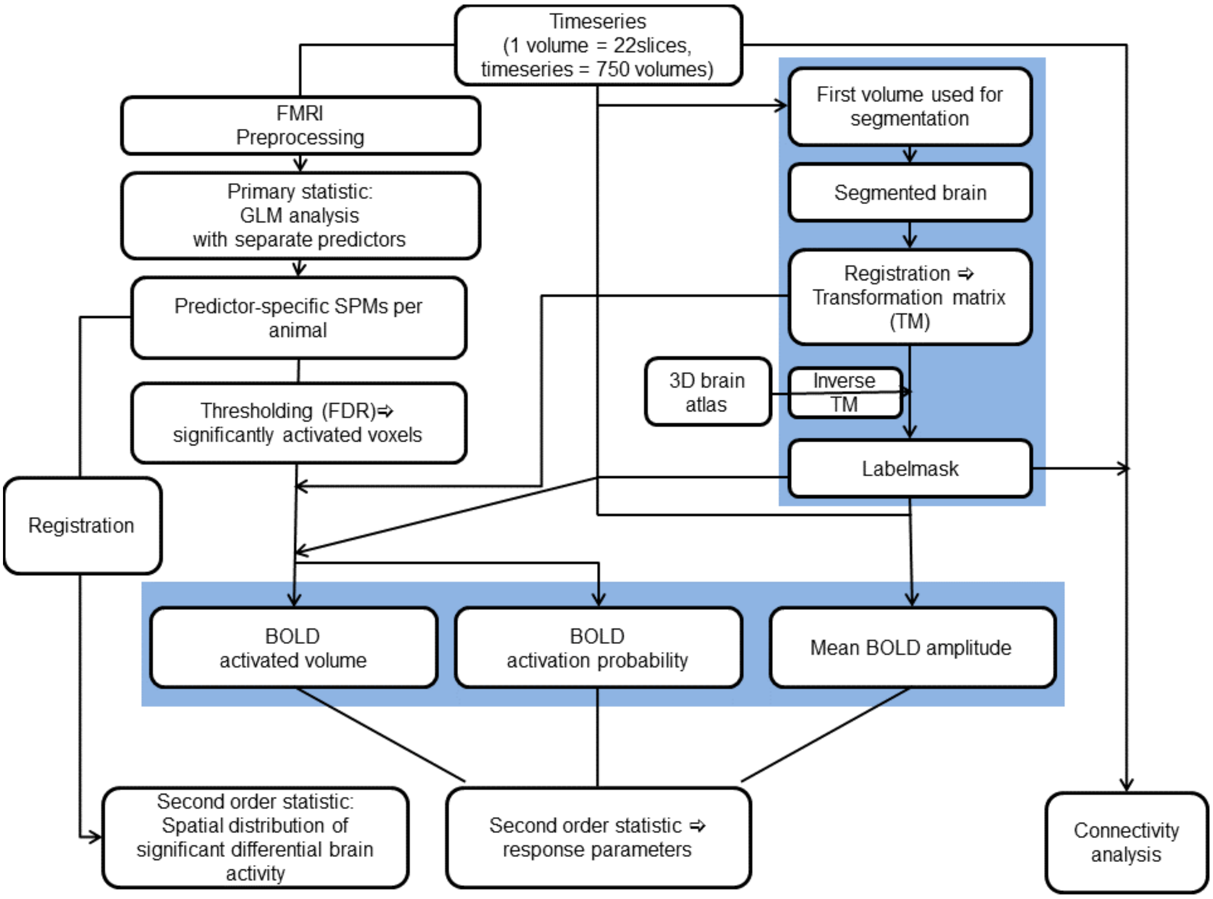

Figure S1: Scheme showing workflow details of the data processing

Displayed are workflow details of the data processing described in detail in the methods section. The blue boxes mark analysis steps per animal besides standard fMRI pre-processing.

Abbreviations:

Acb = nucleus accumbens,

am = amygdala,

amBM = basomedial amygdaloid nucleus

amBL = basolateral amygdaloid nucleus,

amCo = cortical amygdala,

amM = medial amygdaloid nucleus,

BG = basalganglia

BST = bed nucleus of stria terminalis,

cxAss = association cortex,

cxAu = auditory cortex,

cxCg = cingulate cortex,

cxEnt = entorhinal cortex,

cxIns = insular cortex,

cxPdD = dorsal peduncular cortex

cxOrb = orbital cortex

cxPrL = prelimbic cortex,

cxPtA = parietal association cortex,

cxRS = retrosplenial cortex,

cxSens = sensory cortex

cxS1 = primary somatosensory cortex,

cxS1BF = primary sensory cortex barrel field,

cxS1r = primary sensory cortex rest,

cxS2 = secondary somatosensory cortex,

cxVis = visual cortex,

Cl = claustrum,

CoM = corpora mammillaria,

CPu = caudate putamen,

GPL = lateral globus pallidus,

Hb = habenuli,

hcAD = anterior dorsal hippocampus,

hcDGp = posterior layers of the dentate gyrus,

hcPD = posterior dorsal hippocampus,

hcSD = dorsal subiculum,

hcSV = ventral subiculum,

Hip = hippocampus,

HT = hypothalamus,

hyArc = arcuate hypothalamic nucleus,

hyDM = dorsomedial hypothalamus,

hyL = lateral hypothalamus,

hyM = medial hypothalamus,

hyPo = posterior hypothalamus,

hyPV = paraventricular hypothalamic nucleus,

IC = inferior colliculus,

inSens = sensory input,

M1 = primary motor cortex,

M2 = secondary motor cortex,

Mout = motoric output,

outLimb = limbic output,

PAG = periaqueductal gray,

PTA = pretectal area,

PV = paraventricular thalamic nucleus,

SC = superior colliculus,

SN = substantia nigra,

thGL = lateral geniculate nucleus,

ThL = lateral thalamus,

thLD = laterodorsal thalamic nucleus,

thLP = lateral posterior thalamic nucleus,

ThM = medial thalamus,

thMD = mediodorsal thalamus,

thGM = medial geniculate nucleus

thPo = posterior thalamic nuclear group,

thVM = ventromedial thalamic nucleus,

thVL = ventrolateral thalamic nucleus,

thVPM = ventral posteromedial thalamic nucleus,

thVPL = ventral posterolateral thalamic nucleus,

thSM = submedius thalamic nucleus,

TN = tegmantal nuclei,

Red = red nucleus,

VTA = ventral tegmental area,

VP = ventral pallidum,

ZI = zona incerta
